# Supplementary material for: Kauniolide synthase is a P450 with unusual hydroxylation and cyclization-elimination activity
Source: Nat Commun. 2018 Nov 7;9:4657. doi: 10.1038/s41467-018-06565-8 (PMC6220293; doi:10.1038/s41467-018-06565-8)
Supplement: Supplementary file 1 — Supplementary information, corrected.docx [file 41467_2018_6565_MOESM1_ESM.docx]

**Supplementary Information**

**Kauniolide synthase is a P450 with unusual hydroxylation and cyclisation-elimination activity**

Liu et al.,

**Kauniolide synthase is a P450 with unusual hydroxylation and cyclisation-elimination activity**

Qing Liu^†^, Arman Beyraghdar Kashkooli^†^, David Manzano, Irini Pateraki, Lea Richard, Pim Kolkman, Maria Fátima Lucas, Victor Guallar, Ric C.H. de Vos, Maurice C.R. Franssen, Alexander van der Krol, Harro Bouwmeester

^†^: These authors contributed equally to this paper

**Supplementary Figures**

| **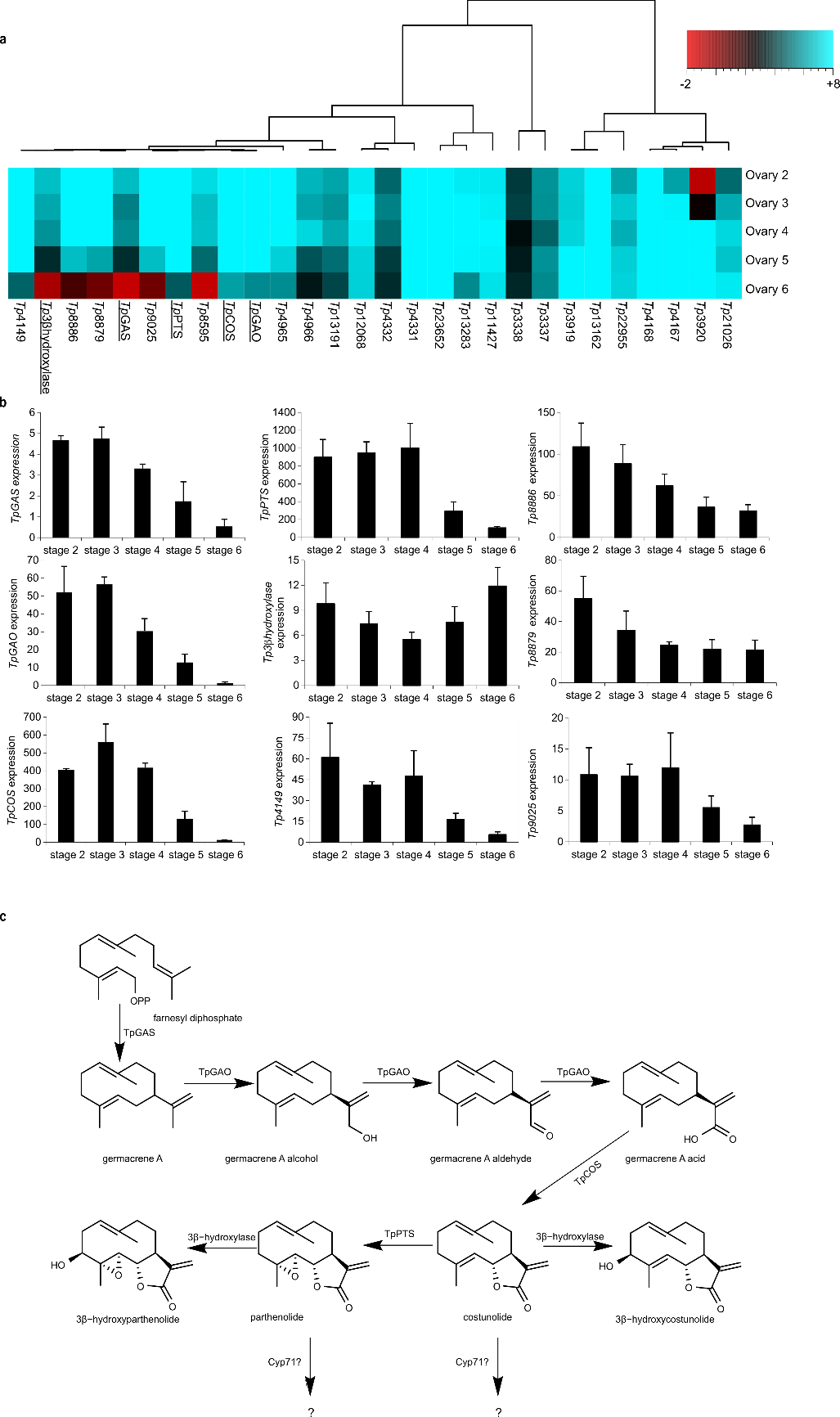** |
| --- |
| **Supplementary Figure 1.** **Expression profile of candidate feverfew CYP71 genes grouped together with characterized feverfew CYP71 genes**. **(a)** Complete linkage cluster analysis of differentially expressed feverfew CYP71 genes. Heat-map representation of the expression data of genes during five developmental stages of feverfew flowers. Each data point is the mean value of the three measured biological replicates. Underlined genes are the characterized TpCYP71 genes. Colour key represents the normalized, log transformed mean gene expression values. Turquoise represents increased, while magenta represents decreased expression. **(b)** Validation of expression of selected obtained genes from RNA-sequencing. Expression profile of candidate feverfew CYP71 genes grouped together with characterized feverfew CYP71 genes (n=3) by Real-Time qPCR. Bars represent means (n = 3) ± standard error (s.e.m.).  **(c)** Proposed feverfew sesquiterpene lactone biosynthesis pathway extension by candidate CYP71 genes. GAS=germacrene A synthase, GAO= germacrene A oxidase, COS= costunolide synthase, PTS= parthenolide synthase. |

| 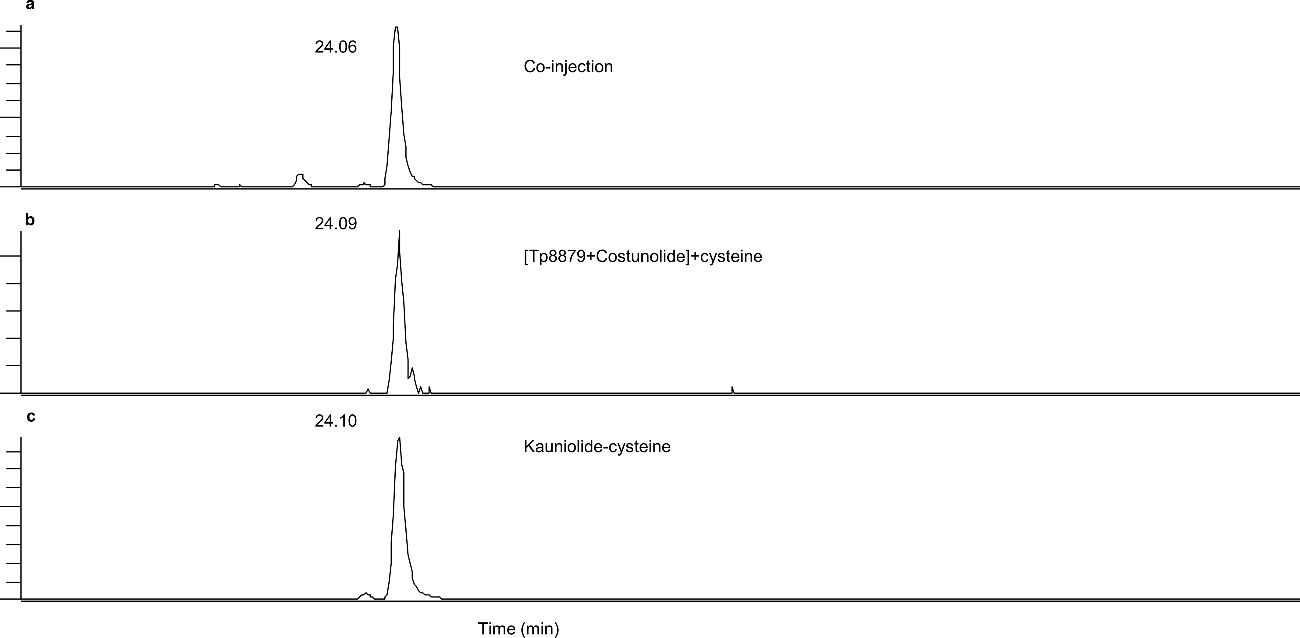 |
| --- |
| **Supplementary Figure 2.** **Chromatogram of LC-Orbitrap-FTMS analysis of enzymatic reaction mixture**. **(a)** co-injection of kauniolide-cysteine and product of feeding costunolide to Tp8879 (TpKLS) incubated with cysteine. **(b)** kauniolide-cysteine production from feeding costunolide to Tp8879 (TpKLS) and subsequent incubated with cysteine **(c)** kauniolide-cysteine. |

| 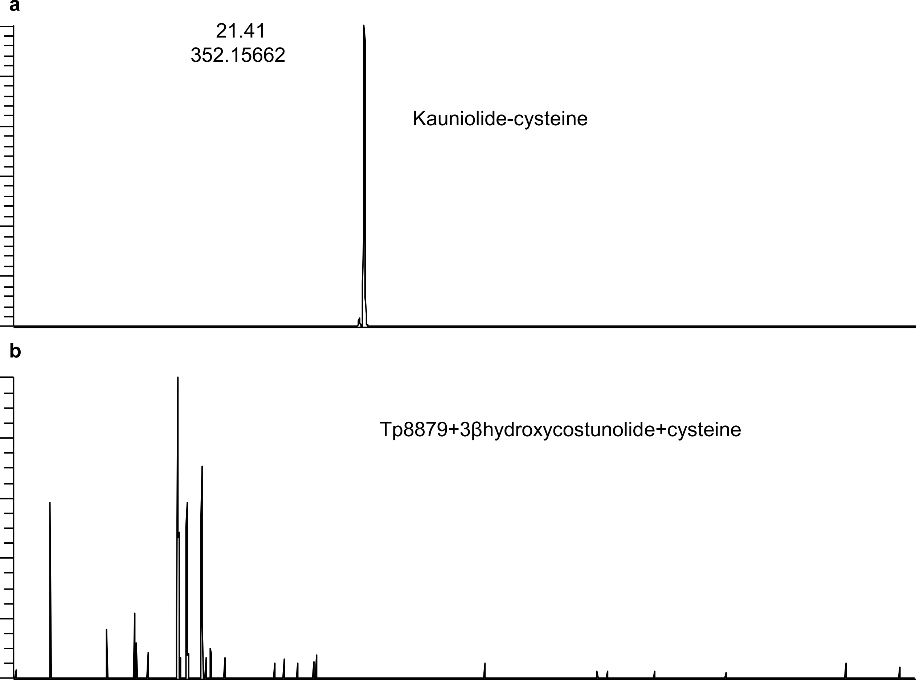 |
| --- |
| **Supplementary Figure 3.** **Feeding 3βhydroxycostunolide to Tp8879 (KLS) does not lead to kauniolide formation. (a)** Chromatogram of enzymatic reaction mixture of kauniolide-cysteine and **(b)** feeding 3β-hydroxycostunolide to Tp8879 (TpKLS) incubated with cysteine. |

|  |
| --- |
| **Supplementary Figure 4.** A view at the active site of the kauniolide synthase model with a costunolide docking pose. C3 of costunolide approaches the iron-oxo species well. Aspartic acid 113 is located ~7Å from C14 of costunolide. A water bridge might be formed within the 7Å. (Asparagine 283 in between aspartic acid and costunolide is also found in the other homology models.) The (walleyed stereo) image was taken in Maestro (Schrödinger). |

| 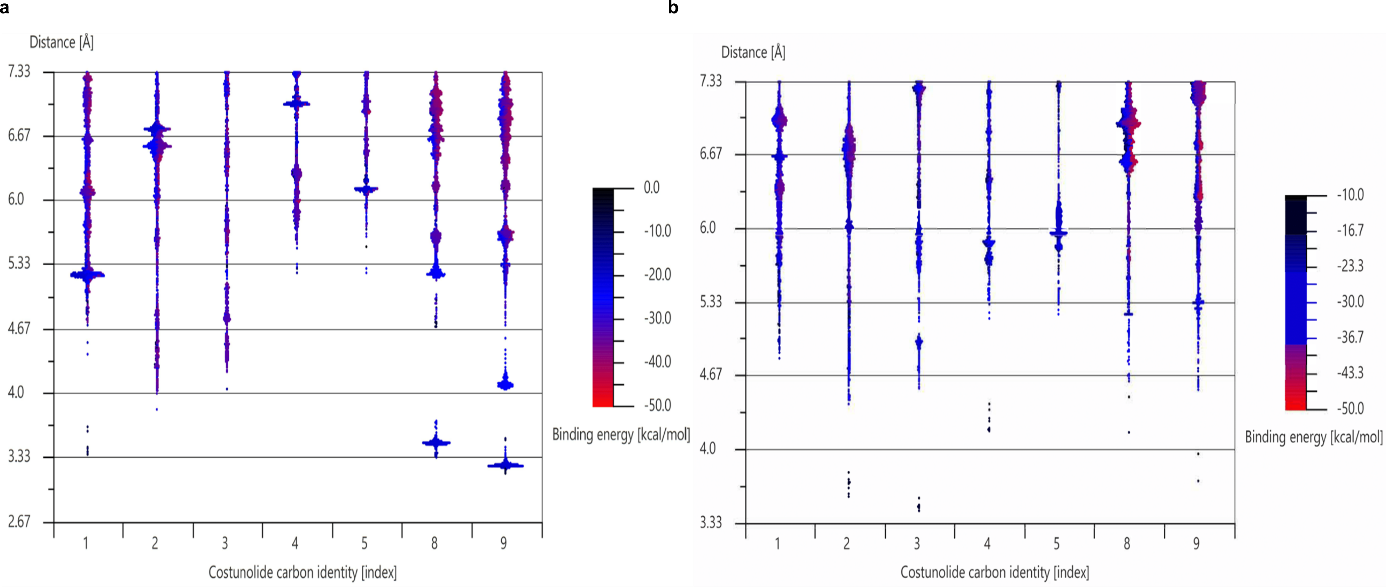 |
| --- |
| **Supplementary Figure 5.** **Plotting preferred docking orientation of costunolide with PELE software.** **(a)** Distance distribution of costunolide carbons relative to the heme-oxyanion in a *Tp*PTS homology model (unconstrained). **(b)** Costunolide carbon distance distribution relative to the heme-oxyanion in a *Tp*PTS homology model (constrained model (15Å)). Colour keys represent the binding energy (kcal mol-1) expressed in negative values. |

| 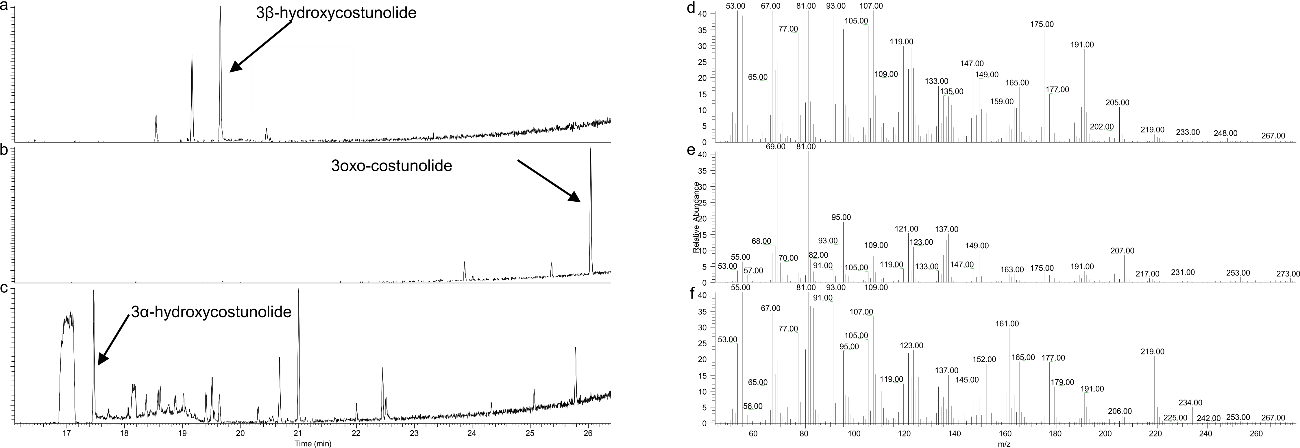 |
| --- |
| **Supplementary Figure 6. Chemical synthesis of 3α-hydroxycostunolide. (a)** GC-MS chromatogram of 3β-hydroxycostunolide. **(b)** GC-MS chromatogram of 3-oxocostunolide, obtained after oxidation with activated MnO_2_ (Sigma). **(c)** GC-MS chromatogram of the reduction product of 3-oxocostunolide, 3α-hydroxycostunolide. The peak at RT = 17.80 is derived from 3α-hydroxycostunolide; note the very broad peak from RT = 17.50-19.50 min, characteristic for on-column Cope rearrangement of some germacrenes (e.g. see ^1^). Right panels represent the in source spectrum of 3β-hydroxycostunolide **(d)**, 3-oxocostunolide **(e)** and 3α-hydroxycostunolide **(f)**. |

| 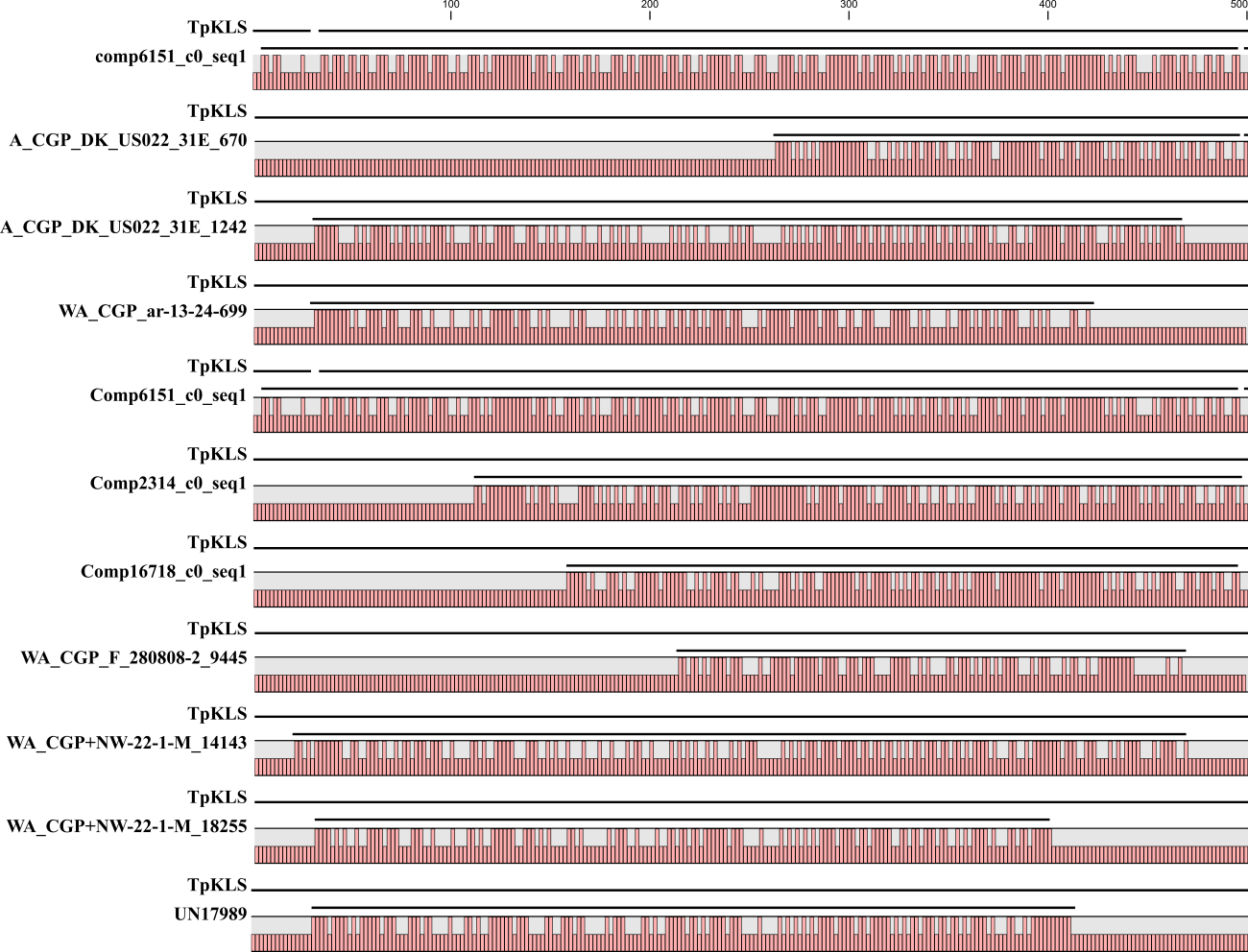 |
| --- |
| **Supplementary Figure 7.** **Homology analysis Asteraceae transcripts** **with *Tanacetum parthenium* kauniolide synthase.** More than 2.3 million Asteraceae transcripts [http://compgenomics.ucdavis.edu/] analysed and highest ranking candidates with TpKLS were individually aligned with TpKLS. |

| 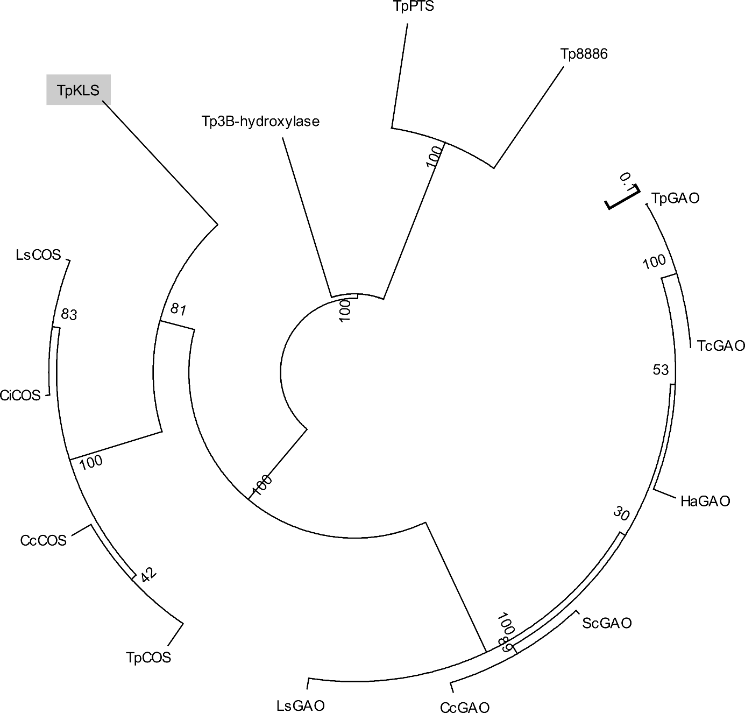 |
| --- |
| **Supplementary Figure 8.** **Phylogenetic tree of CYP71 genes from Asteraceae with known function.** Tp: *Tanacetum parthenium*; Ci: *Cichorium intybus*; Cc: *Cynara* *cardanculus*; Tc: *Tanacetum* *cinerariifolium*; Ls: *Lactuca* *sativa*; Ha: *Helianthus* *annuus* and Sc: *Saussurea* *costus* . Bootstrap values (×10) are from 1000 replicates. |

| 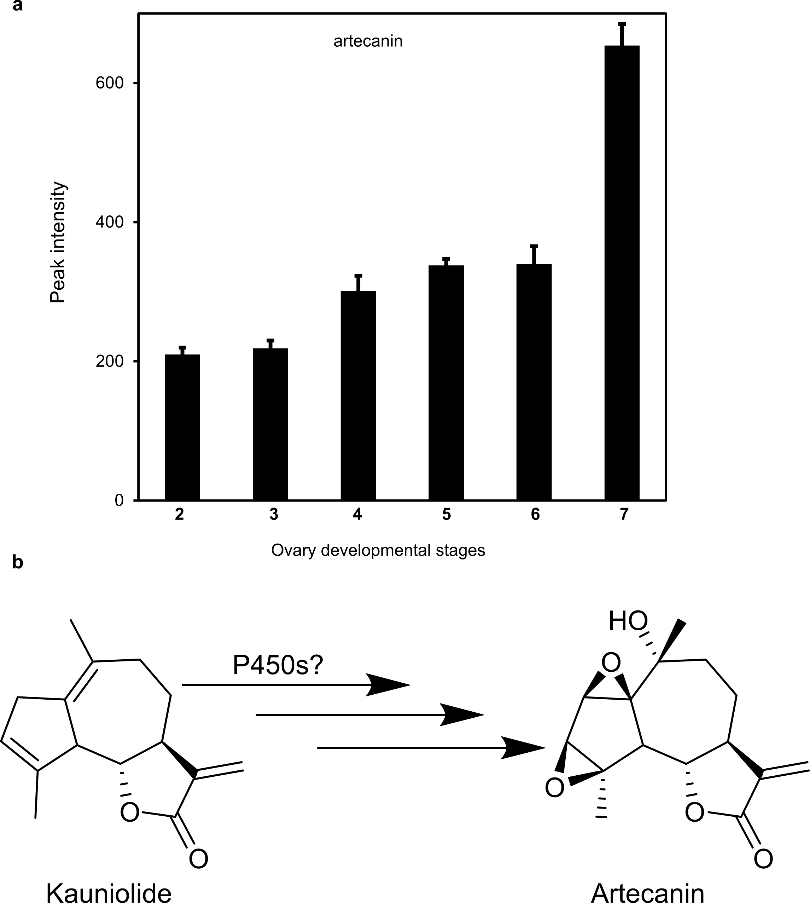 |
| --- |
| **Supplementary Figure 9.** **Tp8886 adds an oxygen group to kauniolide which might be an intermediate in artecanin biosynthetic pathway. (a)** Accumulation pattern of artecanin during ovary developmental stages of feverfew flowers. Bars represent means (n = 3) ± s.e.m. **(b)** Proposed biosynthetic pathway of artecanin. Several P450 enzymes would be involved in biosynthesis of artecanin from kauniolide. |

**Supplementary Tables**

| **Supplementary Table 1**. **Contig names and Asteraceae species from which the contigs were selected in Supplementary Figure 6.** | |
| --- | --- |
| **Contig name** | **Plant species** |
| comp6151_c0_seq1 | *Cichorium endivia* Collection ID CHE-3178 |
| A_CGP_DK_US022_31E_670 | *Centaurea diffusa* Collection ID DK_US022-31E |
| A_CGP_DK_US022-31E_1242 | *Centaurea diffusa* Collection ID DK_US022-31E |
| WA_CGP_AR-13-24_669 | *Centaurea solstitialis* Collection ID AR-13-24 |
| Comp2314_c0_seq1 | *Leontodon* |
| Comp16718_c0_seq1 | *Leontodon* |
| WA_CGP_F_280808-2_9445 | *Cirsium arvense* Collection ID 280808-2 |
| WA_CGP+NW-22-1-M_14143 | *Cirsium arvense* Collection ID 280808-2 |
| WA_CGP+NW-22-1-M_18255 | *Cirsium arvense* Collection ID 280808-2 |
| UN17989 | *Cynara cardunculus* Collection ID ATL |

**Supplementary Table 2**. **Query – template alignments where the homology models have been built from.**

| **Query (seq. identity; coverage)** | **Final query-template alignment** |
| --- | --- |
| **Kauniolide synthase**  **(18% I; 95% C)** | **Kauniolide Synthase, 495 residues**  MALYITFLFIVSSLVLFYFFVLNQKPKGKLPPGPPKLPIIGNIPQVAGKLPHHVLRDLARKYGPVMHLQLGHLSTIVVSSPRLAEHVLKTNDLAVSNRPYSLVGDVVLYGGSDVVF~~GNYGDYWRQMKKIMTTEALSA~~~~~~KKVREFSGIRDHEINNMIEFIRSTLGKPFHLREGVMQRNNNIICKALFGDHSKQQ~~~DLLIEIVEELVVLASGFQLADFFP~~~~~KLKFLTAISGMKSKLTKVHNELDNIFDELFRERKIKRQT~NGATEDDLLD~VLFNIKER~~~~GGLQFPIEDNNIKAIFVNMFIGGTDTSVVTIEWTMTQMMRFPEVMKKAQAEVRRVFKGKQTITEKDLEQLVYLRCVVKEALRLYAPIPILLPRESREKFQIDGYDIPVGTRVLVNAYACSTDPEYWDDADSFKPERFEKSAVDFMGRNYEYLPFGTGRRICPGITFGLNVAEIIIAKLIYHFDWELPNGLSPKDIDLSENFGVVADKKVPLEIIPTRYYPMS  **2PQ5_A, 471 residues**  ~~~~~~~~~~~~~~~~~~~~~~~~SSKGKLPPGP*TP*LPFIGNYLQLNTEQMYNSLMKISERYGPVFTIHLGPRRVVVLCGHDAVREALVDQAEEFSGRGEQATFDWV~FKGYGVVFSN~~~GERAKQLRRFSIATLRDFGVGKRG~~~~~IEERIQEEAGFLIDALRGTGGANIDPTFFLSRTVSNVISSIVFGDRFDYKDKE~~~~FLSLLRMMLGIFQFTSTSTGQLYEM~~~FSSVMKHLPGPQQQAFQLLQGLEDFIAKKVEHNQRTLDPNSPRDFIDSFLIRMQEEEKNP~~~NTEFYLKNLVMTTLQLFIGGTETVSTTLRYGFLLLMKHPEVEAKVHEEIDRVIGKNRQPKFEDRAKMPYMEAVIHEIQRFGDVIPMSLARRVKKDTKFRDFFLPKGTEVYPMLGSVLRDPSFFSNPQDFNPQHFLNEKGQF~KKSDAFVPFSIGKRNCFGEGLARMELFLFFTTVMQNFRLKSSQSPKDIDVSPKHVGFATIPRNYTMSFLPRXHHH |
| **3β-Hydroxylase (95% I; 95% C)** | **3β-Hydroxylase, 499 residues**  MFSSFETLILSFVSLFFMMIFIHSKWISSYSKMAKNLPPSPFGLPIIGNLHQLGM*TP*Y~NSLRTLAHKYGSLMLIHLGSVPVIVASSAEAAQEIMKTHDQIFSTRPKMNIASIVSFDAKIVAFSPYGEHWRQSKSVYLLNLLSTKR~VQSFRHVREDETNLMLDVIENSCGSEIDLSNMIMSLTNDVVCRIAYGRKY~~~~~Y~EDWFKELMKEVMDVLGVFSVGNYVPSLSWIDRLSGLEGRAYKAAKQLDAFLEGVVKQHETKSNESMRDQDVVDILLETQREQASAG*TP*FHRDTLKALMQEMFIAGTDTTSTAIEWEISEVIKHPRVMKKLQQELDEIAQGRQRITEEDLEDTQHPYLEAILKESMRLHIPVPLLLPREATHDVKVMGYDIAAGTQVLINAWMIARDPTIWEDADEFKPERFLDTNIDYKGLNFELLPFGAGRRGCPGIQFAMSVNKLALANLVYKFDFKLPNGLRLEQLDMTDSTGITVRRKYPLLVIPTARF  **1NR6_A, 472 residues**  ~~~~~~~~~~~~~~~~~~~~~~~~~~MAKKTSSKGKLPPGP*TP*FPIIGNILQIDAKDISKSLTKFSECYGPVFTVYLGMKPTVVLHGYEAVKEALVDLGEEFAGRGSVPILEKVS~KGLGIAFS~NAKTWKEMRRFSLMTLRNFGMGKRSIEDRIQEEARCLVEELRKTNASPCDPTFILGCAPCNVICSVIFHNRFDYKDE~EFLKLMESLHENVELLG*TP*WLQVYNNFPALLDYFPGIHKTLLKNADYIKNFIMEKVKEHQKL~LDVNNPRDFIDCFLIKMEQENNL~~EFTLESLVIAVSDLFGAGTETTSTTLRYSLLLLLKHPEVAARVQEEIERVI~GRHRSPCMQDRSRM~PYTDAVIHEIQRFIDLLPTNLPHAVTRDVRFRNYFIPKGTDIITSLTSVLHDEKAFPNPKVFDPGHFLDESGNFKKSDY~FMPFSAGKRMCVGEGLARMELFLFLTSILQNFKLQSLVEPKDLDITAVVNGFVSVPPSYQLCFIPIHHH |
| **Parthenolide Synthase**  **(26% I; 93% C)** | **Parthenolide Synthase, 506 residues**  MDTSTSFPSLFLPTLCTILISYIIIKYVLIWNRSSMAAFNLPPSPPKLPIIGNIHHVFSKNVNQTLWKLSKKYGPVMLIDTGAKSFLVVSSSQMAMEVLKTHQEILSTRPSNEGTKRLSYNFSDITFSPHGDHWRDMRKVFV~~~NEFLGPKRAGWFNQVLRMEIKDVINNLSSNPLNTSINLNEMLLSLVYRVVCKFAFGKSYREEPFNGVTLKEMLDESMVVLAGSSADMFPTFGWILDKLYGWNDRLEKCFGNLDGFFEMIINEHLQSASETSEDEKDFVHS~LVELSL~KDPQFTKDYIKALLLNV~~LLGA~IDTTFTTIVWAMSEIVKNTQVMQKLQTEIRSCIGRKEEVDATDLTNMAYLKMVIKETLRLHPPAPLLFPRECPSHCKIGGYDVFPGTCVVMNGWGIARDPNVWKEIPN~~EFYPERFENFNIDFLGNHCEMIPFGAGRRSCPGMKSATSTIEFTLVNLLYWFDWEVPSGMNNQDLDME~~EDGFLVIQKKSPLFLIPIKHI  **4GQS_A, 470 residues**  ~~~~~~~~~~~~~~~~~~~~~~~~~~~~~~~~~~SSGRGKLPPGP*TP*LPVIGNILQIDIKDVSKSLTNLSKIYGPVFTLYFGLERMVVLHGYEVVKEALIDLGEEFSGRGHFPLAERANRGFG~IVFS~NGKRWKEIRRFSLMTLRNFGMGKRS~~IEDRVQEEARCLVEELRKTKAS~PCDPTFILGCAPCNVICSIIFQKRFDYKDQQFLNLMEKLNENIRIVS*TP*WIQICNNFPTIIDYFPGTHNKLLKNLAFMESDILEKVKEHQESMD~~INNPRDFIDCFLIKMEKEKQNQQSEFTIENLVITAADLLGAGTETTSTTLRYALLLLLKHPEVTAKVQEEIERVVGRNRSPCMQDRGHMPYTDAVVHEVQRYIDLIPTSLPHAVTCDVKFRNYLIPKGTTILTSLTSVLHDN~~~KEFPNPEMFDPRHFLDEGGNFKKSNY~FMPFSAGKRICVGEGLARMELFLFLTFILQNF~~NLKSLIDPKDLDT*TP*VVNGFASVPPFYQLCFIPIHH~ |

**Supplementary Reference**

1 de Kraker, J.-W., Franssen, M. C. R., de Groot, A., König, W. A. & Bouwmeester, H. J. (+)-Germacrene A Biosynthesis : The Committed Step in the Biosynthesis of Bitter Sesquiterpene Lactones in Chicory. *Plant Physiology* **117**, 1381-1392 (1998).
